# Q Fever Endocarditis in a Saudi Child: A Case Report and Literature Review

**DOI:** 10.7759/cureus.6322

**Published:** 2019-12-08

**Authors:** Alhussain Alzahrani, Turki Alqarni, Mohammed Alsalmi, Abdullah Ashi, Rahaf Waggass

**Affiliations:** 1 Miscellaneous, College of Medicine, King Saud Bin Abdulaziz University for Health Sciences, Jeddah, SAU; 2 Pediatrics, College of Medicine, King Saud Bin Abdulaziz University for Health Sciences, Jeddah, SAU; 3 Cardiology, King Faisal Cardiac Center, King Abdulaziz Medical City, Jeddah, SAU

**Keywords:** coxiella burnetii, q fever, endocarditis, male, heart defects, saudi arabia

## Abstract

Q fever is a zoonotic disease that is caused by Coxiella burnetii, a gram-negative coccobacillary bacterium. Human infection primarily occurs following the inhalation of aerosols containing C. burnetii. The infection can either present in an acute or chronic form. The three main presentations are flu-like syndrome, atypical pneumonia, and hepatitis. Chronic Q fever mainly affects the heart where the disease manifests as endocarditis. In this case report, the patient was born at term with congenital heart defects, namely double outlet right ventricle (DORV), ventricular septal defects (VSD), and coarctation of the aorta. He underwent coarctation repair and pulmonary artery binding. At the age of three years, he presented with palpitation, sudden high-grade fever, myalgia, and dyspnea. Endocarditis was suspected due to a history of a surgical repair of congenital heart defects. Blood cultures were negative, however, a diagnosis of Q fever endocarditis was confirmed based on serologic titers.

Q fever endocarditis is a challenging diagnosis since the echocardiography findings are often nonspecific. Moreover, Q fever can present as negative-culture endocarditis with low sensitivity of blood and tissue polymerase chain reaction (PCR) for C. burnetii. Hence, the modified Duke criteria has considered phase 1 immunoglobulin G (IgG) titers of 1:800 or more as diagnostic for infective endocarditis. Although uncommon, physicians should maintain a high index of suspicion for Q fever endocarditis, especially among patients with pre-existing structural heart disease and associated symptoms and risk factors such as animal exposure.

## Introduction

Q fever is a zoonotic disease that is caused by Coxiella burnetii, a gram-negative coccobacillary bacterium. This organism typically infects certain domestic animals such as goats, sheep, and cattle [1]. The most common site of high concentrations of the organism is the placenta and amniotic fluid, though it can also be present in urine, feces, and milk of the infected animals [2]. Human infection primarily occurs following the inhalation of aerosols containing C. burnetii. Other rarer modes of disease transmission are through ingestion of raw milk, tick bite, and person-to-person transmission. This infection can either present as an acute form, where the antibody response to C. burnetii phase II antigen is predominant and is higher than the response to the phase I antigen, or it can present in a chronic form after months or years of the initial infection and it is associated with a rising phase I immunoglobulin G (IgG) titer. The acute illness has an estimated incubation period of one to three weeks. This phase presents with various clinical features that vary from patient to another. The three main presentations are flu-like syndrome, atypical pneumonia, and hepatitis [3]. Moreover, symptomatic patients might be ill for weeks or months if untreated. The chronic Q fever mainly affects the heart where the disease is manifested as endocarditis in 60%-80% of all cases of chronic Q fever worldwide [4]. It occurs in up to 5% of all infected humans and is associated with a high mortality rate if left untreated [5]. In Saudi Arabia, there have been five reported cases of endocarditis due to Q fever [6-9]. In this case, we present an 8-year-old male patient who was diagnosed with endocarditis as part of chronic Q fever, along with a detailed review of the previously reported cases.

## Case presentation

**Figure 1:**
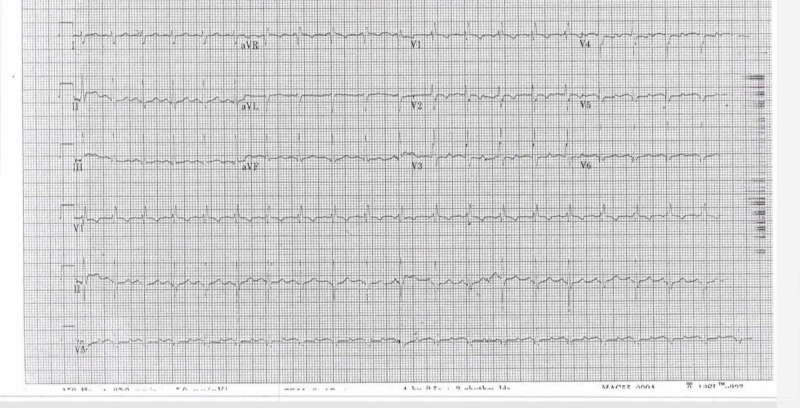
ECG showing normal sinus rhythm, right axis deviation, RSR pattern in V1 indicating RBBB, possible left ventricular hypertrophy, and nonspecific T wave abnormality ECG: electrocardiogram; RSR: regular sinus rhythm; RBBB: right bundle-branch block.

An 8-year-old boy, who is the first child of healthy consanguineous first-cousin Saudi parents, underwent coarctation repair and pulmonary artery binding soon after birth, followed by Yasui procedure with right ventricle to pulmonary artery (RV-PA) conduit size 14 mm. Afterwards, he was being followed up with the diagnosis of double outlet right ventricle (DORV), ventricular septal defects (VSD), and coarctation of the aorta at the National Guard Hospital - Jeddah, Saudi Arabia. At the age of three years, the patient presented to the emergency room with palpitation, high-grade fever reaching 40^o ^C that lasted for two months, myalgia, and dyspnea. Therefore, he was admitted to the pediatrics department. The family lives on a farm with exposure to animals. The father is a soldier and the mother is a housewife. There is also a history of goat milk ingestion. On physical examination, the patient was oriented and not in respiratory distress. There were no signs of dehydration, jaundice or rash. Throat examination showed enlarged tonsils that were not congested. Chest auscultation revealed bilateral clear, equal air entry with no added sounds. Cardiovascular exam revealed that there was a median sternotomy scar; S1 and 2 with pan-systolic murmur. His electrocardiogram (ECG) showed normal sinus rhythm, right axis deviation, RSR pattern in V1 indicating right bundle-branch block (RBBB), possible left ventricular hypertrophy, and nonspecific T wave abnormality (Figure 1). His echocardiography showed no residual VSD patch leak, patent Damus-Kaye-Stansel (DKS) anastomosis, no residual coarctation of aorta, good biventricular systolic function, mild mitral and tricuspid valve insufficiency, and mild supra-valvular conduit stenosis at conduit anastomosis with distal main pulmonary artery. Moreover, large vegetation was also observed. Abdominal ultrasound revealed hepatosplenomegaly. Neurological examination was grossly intact and developmental milestones were appropriate for his age.

Laboratory tests revealed white blood cells (WBC): 10900/mm3 (lymphocyte 5.69, neutrophil 3.5), hemoglobin: 12.9 gr/ld, platelets: 284 x 103/mm3. Hepatic function test was normal. C-reactive protein was 24 mg/dl and erythrocyte sedimentation rate was found to be 14; Epstein-Barr virus (EBV) IgM was positive. Cytomegalovirus (CMV), multiple blood and urine cultures were all negative. Viral serology for toxoplasma was negative. Chest X-ray was normal. Q fever endocarditis was suspected due to persistent high-grade fever, hepatosplenomegaly and the fact that the patient had cardiac abnormalities as well as animal exposure. Therefore, Q fever infectious serology was ordered. Meanwhile, the patient was started on vancomycin 180 mg Q6hrs and gentamicin 15 mg Q12hrs; gentamicin was discontinued after 15 days. The patient was recommended to complete the vancomycin for four weeks. After four weeks, the echo was repeated and still there was small vegetation. The Infectious Disease team recommended completing it for two more weeks. Later on, Q fever infectious serology revealed that C. burnetii phase II IgG titer was measured to be 1:64000+, IgM titer was measured to be 1:4096+, phase I IgG titer was 1:16000+, IgM phase I was >1:64000+ and this was compatible with chronic infection.

The diagnosis of infective endocarditis was confirmed and the treatment was switched to oral doxycycline with the plan of at least 18 months of therapy. However, ciprofloxacin was added on later as the patient had a minimum response to doxycycline and high liver function tests. The patient is doing well and is responding to this treatment.

## Discussion

Q fever infection can result from inhalation, ingestion or contact with contaminated farm animals with C. burnetii infection [10]. Negative-culture Q fever endocarditis can develop following months or years of acute Q fever infection [11]. The initial presentation includes unexplained illness with low-grade fever in patients with aneurisms, valvular heart disease or vascular prosthesis [12]. In 1937, Q fever was recognized as a new clinical entity [13]. Thirty years later, Q fever was recognized as a public health problem in Saudi Arabia [7]. To our knowledge, there are five Saudi cases of Q fever published in the literature [6-9]. Two cases were pediatrics - ages 11 and 13 years; they presented with Q fever endocarditis in pre-existing valvular disease. The index patient presented at three years old with Q fever endocarditis.

Q fever endocarditis is a challenging diagnosis since the ECG findings are often nonspecific. In addition, Q fever can present as negative-culture endocarditis with sensitivity of blood and tissue PCR for C. burnetii of 50%-60% [14]. Hence, the modified Duke criteria has considered phase I IgG titers of 1:800 or more as diagnostic for infective endocarditis [15]. However, clinical correlation with a serological test is important to establish the diagnosis. Guideline from the Dutch Q Fever Consensus Group considers proven Q fever diagnosis if positive blood or tissue PCR of C. burnetii or phase I IgG titers ≥1:800, positive endocarditis in the modified Duke criteria or imaging studies of a large vessel or prosthetic infection [16]. However, the diagnosis of chronic Q fever endocarditis can be extremely difficult because vegetative lesions are visualized by echocardiography in approximately 12% of patients [17]. Our patient had phase I IgG antibody titer >1:800 for C. burnetii and evidence of endocardial involvement which is the large vegetation that was detected on echocardiogram.

The optimum treatment of Q fever endocarditis includes doxycycline (100 mg/bid) and hydroxychloroquine (200 mg/bid) for the duration of 24 months. Furthermore, it is recommended to establish a goal of therapy of phase IgG titers less than 1:800 by maintaining doxycycline serum concentration 5 g/ml. Since photosensitivity and eye toxicity is problematic in the Q fever regimen in the pediatric popultion, literature recommended alternative treatments. Children younger than eight-year-old with Q fever endocarditis can be given trimethoprim/sulfamethoxazole as an alternative therapy of doxycycline and hydroxychloroquine. A case of three-year-old boy with intractable Q fever that was unresponsive to several antimicrobial therapies, has improved after the addition of interferon gamma three times a week [18]. The index patient was treated with doxycycline 35 ml twice daily. However, ciprofloxacin was added on later as the patient had a minimum response to doxycycline and high lever function test.

The prevalence of Q fever endocarditis in Saudi Arabia remains undetermined. In a study conducted in the central region of Saudi Arabia, on patients with fever of unknown origin, 18 (35.2%) patients out of 51 were positive for phase II C. burnetii specific IgG antibodies [19]. It is important to consider the diagnosis of Q fever endocarditis in patients with chronic fever and history of cardiac surgery. This case adds to the existing limited literature in Saudi Arabia regarding Q fever endocarditis as the third pediatric reported case.

## Conclusions

In conclusion, Q fever endocarditis is a challenging diagnosis since the ECG findings are often nonspecific. Moreover, Q fever can present as negative-culture endocarditis with sensitivity of blood and tissue PCR for C. burnetii of 50%-60%. Hence, the modified Duke criteria has considered phase 1 IgG titres of 1:800 or more as diagnostic for infective endocarditis. This case reaffirms that physicians should maintain a high index of suspicion of Q fever infective endocarditis, especially among patients with pre-existing structural heart disease and associated risk factors.

## References

[1] Gürtler L, Bauerfeind U, Blümel J (2014). Coxiella burnetii - pathogenic agent of Q (query) fever. Transfus Med Hemother.

[2] Anderson A, Bijlmer H, Fournier PE (2013). Diagnosis and management of Q fever—United States, 2013: recommendations from CDC and the Q Fever Working Group. MMWR Morb Mortal Wkly Rep.

[3] Honarmand H (2012). Q fever: an old but still a poorly understood disease. Interdiscip Perspect Infect Dis.

[4] Million M, Thuny F, Richet H, Raoult D (2010). Long-term outcome of Q fever endocarditis: a 26-year personal survey. Lancet Infect Dis.

[5] Maurin M, Raoult D (1999). Q fever. Clin Microbiol Rev.

[6] Saginur R, Silver S, Bonin R, Carlier M, Orizaga M (1985). Q-fever endocarditis. Can Med Assoc J.

[7] Ross P, Jacobson J, Muir J (1983). Q fever endocarditis of porcine xenograft valves. Am Heart J.

[8] Angelakis E, Johani S, Ahsan A, Memish Z, Raoult D (2014). Q fever endocarditis and new Coxiella burnetii genotype, Saudi arabia. Emerg Infect Dis.

[9] Al-Hajjar S, Hussain Qadri S, Al-Sabban E, Jäger C (1997). Coxiella burnetii endocarditis in a child. Pediatr Infect Dis J.

[10] Fournier PE, Marrie TJ, Raoult D (1998). Diagnosis of Q fever. J Clin Microbiol.

[11] Brouqui P, Dupont HT, Drancourt M (1993). Chronic Q fever. Ninety-two cases from France, including 27 cases without endocarditis. Arch Int Med.

[12] Houpikian P, Raoult D (2005). Blood culture-negative endocarditis in a reference center: etiologic diagnosis of 348 cases. Medicine.

[13] Derrick EH (1983). Q fever, a new fever entity: clinical features, diagnosis and laboratory investigation. Rev Infect.

[14] Kampschreur LM, Wegdam-Blans MCA, Wever PC (2015). Chronic q fever diagnosis—consensus guideline versus expert opinion. Emerg Infect Dis.

[15] Li J, Sexton D, Mick N (2000). Proposed modifications to the Duke criteria for the diagnosis of infective endocarditis. Clin Infect Dis.

[16] Wegdam-Blans MCA Kampschreur LM, Delsing CE (2012). Chronic Q fever: review of the literature and a proposal of new diagnostic criteria. J Infect.

[17] Gunn TM, Raz GM, Turek JW, Farivar RS (2013). Cardiac manifestations of Q fever infection: case series and a review of the literature. J Card Surg.

[18] Morisawa Y, Wakiguchi H, Takechi T, Kurashige T, Nagaoka H (2001). Intractable Q fever treated with recombinant gamma interferon. Pediatr Infect Dis J.

[19] Almogren A, Shakoor Z, Hasanato R, Adam MH (2013). Q fever: a neglected zoonosis in Saudi Arabia. Ann Saudi Med.

